# Synthesis and Evaluation of Graphene Aerogel‐Supported Mn_x_Fe_3−x_O_4_ for Oxygen Reduction in Urea/O_2_ Fuel Cells

**DOI:** 10.1002/open.201900105

**Published:** 2019-05-14

**Authors:** Keyru Serbara Bejigo, Bang Ju Park, Ji Hyeon Kim, Hyon Hee Yoon

**Affiliations:** ^1^ Department of Chemical and Biological Engineering Gachon University 1342 Seongnam-daro Seongnam S. Korea; ^2^ Department of Electronic Engineering Gachon University 1342 Seongnam-daro Seongnam S. Korea

**Keywords:** oxygen reduction reaction, urea fuel cell, anode catalyst, manganese ferrite, graphene aerogel

## Abstract

Graphene aerogel‐supported manganese ferrite (Mn_x_Fe_3−x_O_4_/GAs) and reduced‐graphene oxide/manganese ferrite composite (MnFe_2_O_4_/rGO) were synthesized and studied as cathode catalysts for oxygen reduction reactions in urea/O_2_ fuel cells. MnFe_2_O_4_/GAs exhibited a 3D framework with a continuous macroporous structure. Among the investigated Fe/Mn ratios, the more positive oxygen reduction onset potential was observed with Fe/Mn=2/1. The half‐wave potential of MnFe_2_O_4_/GAs was considerably more positive than that of MnFe_2_O_4_/rGO and comparable with that of Pt/C, while the stability of MnFe_2_O_4_/GAs significantly higher than that of Pt/C. The best urea/O_2_ fuel cell performance was also observed with the MnFe_2_O_4_/GAs. The MnFe_2_O_4_/GAs exhibited an OCV of 0.713 V and a maximum power density of 1.7 mW cm^−2^ at 60 °C. Thus, this work shows that 3D structured graphene aerogel‐supported MnFe_2_O_4_ catalysts can be used as an efficient cathode material for alkaline fuel cells.

## 1 Introduction

Recently, anion exchange membrane fuel cells (AEMFCs) have received considerable attention in area of fuel technology with promising output. AEMFCs have benefits over proton exchange membrane fuel cells (PEMFCs) as operated in alkaline media, which boosts oxygen reduction kinetics and allows the use of non‐precious metal catalysts.^1^ Other benefits of AEMFCs are lower fuel cross‐over due to the movement of anions against fuel and fuel flexibility;^2^ various fuels such as H_2_, methanol, ethanol, and glucose can be used in AEMFCs. Urea (CO(NH_2_)_2_), is an industrial product mainly used as an agricultural fertilizer, can also be used as a fuel in AEMFCs. Urea is a non‐toxic, non‐flammable, and biodegradable compound, and is relatively cheap and convenient to store and transport compared with hydrogen.^3^ Furthermore, urine and urea‐containing wastes can be purified with electricity generation using AEMFCs.

In AEMFCs, anode reaction oxidizes the fuel with the release of electrons, which pass through an external circuit, while the electrolyte membrane allows the transfer of OH^−^ produced from the oxygen reduction reaction (ORR) at the cathode.^4^ ORR is known to be multifaceted owing to its multistep and multi‐electron transfer behavior involving numerous adsorption/desorption stages for oxygen‐containing species such as O, O_2_^−^, OH, HO_2_^−^, and H_2_O_2_ as reaction intermediates, which makes it slower.^5^,^6^ Currently, Pt is the most active ORR catalyst. However, its high cost is a critical barrier for practical implementation. To reduce Pt consumption, it has been alloyed with non‐precious metals such as Co, Cr, and Ni, which are reported to be efficient catalyst for ORR.^7^ As an alternative to Pt, non‐noble catalysts for ORR including transition metal oxides,^8^ transition metal nitrides,^9^ and their chalcogenides^10^ have been studied and reported as promising catalysts for ORR. Among the new approaches, oxides of transition metals exhibited outstanding performance for ORR.^11^ For instance, manganese ferrite (MnFe_2_O_4_), which has an inverse spinel structure with multiple valance electrons, has been proved to be a good ORR catalyst in alkaline media. Zhu and coworkers also reported that manganese‐substituted ferrite outperformed over others (Cu‐ and Co‐substituted ferrite) and was even comparable to Pt in basic media.^12^ However, MnFe_2_O_4_ is a semi‐conductive material that leads to insufficient performance resulting from poor ion and electron transfer.^13^ The catalytic activity of MnFe_2_O_4_ was improved by integrating it with other materials that are capable of boosting conductivity in addition to the reduction in agglomeration of active catalyst. MnFe_2_O_4_‐supported conductive materials such as graphene and polyaniline composites were studied for ORR and exhibited higher catalytic activity than MnFe_2_O_4_ did.^14^

Graphene is one of the carbon‐based nanomaterials with a high electrical conductivity, large surface area, and good mechanical strength, which make it an ideal support for catalyst materials. Incorporating metal and their oxide nanoparticles into graphene creates porous networks that enhance both catalyst activity and its stability.^15^,^16^ Graphene‐supported transition metal oxides such as MnCo_2_O_4_ and Mn_3_O_4_ nanoparticles exhibited good ORR performance in alkaline media.^17^,^18^ Recently, graphene aerogel having a three‐dimensional mesoporous structure has attracted the most attention owing to its high surface area, light weight, and high porosity, which allow sufficient electron transfer pathways.^19^, ^20^, ^21^ Wang et al. developed ferric oxide on a graphene aerogel for ORR, which outperformed over commercial Pt/C.^22^

In this study, a manganese ferrite‐decorated graphene aerogel (Mn_x_Fe_3−x_O_4_/GAs) composite was synthesized by a reducing agent‐assisted hydrothermal self‐assembly process and was studied as a cathode material in a urea/O_2_ fuel cell. The structural and morphological properties of the Mn_x_Fe_3−x_O_4_/GAs catalyst were characterized. The electrochemical activity of the Mn_x_Fe_3−x_O_4_/GAs‐modified electrodes were studied towards ORR using cyclic voltammetry and linear sweep voltammetry.

In addition, the performances of urea/O_2_ fuel cells comprising MnFe_2_O_4_/GAs as a cathode material was evaluated.

## 2 Results and Discussion

### 2.1 Characterization of GO, MnFe_2_O_4_, MnFe_2_O_4_/rGO, and MnFe_2_O_4_/GAs

**Figure 1:**
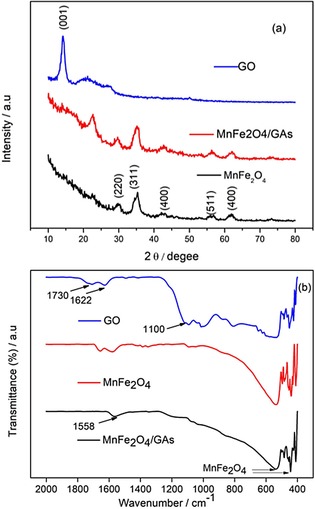
XRD patterns (a) and FTIR spectra (b) of GO, MnFe_2_O_4_, and MnFe_2_O_4_/GAs.

The structures and crystallographic phases of graphene oxide (GO), MnFe_2_O_4_, and MnFe_2_O_4_/GAs particles were studied by XRD as plotted in Figure 1a. The pristine GO showed a characteristic reflection peak corresponding to the (001) plane. This peak originates from the inter planner spacing of graphene oxide due to the presence of different oxygenated functionalities on the surface. In the XRD pattern of MnFe_2_O_4_/GAs (Figure 1a) and Mn_0.5_Fe_2.5_O_4_/GAs (Suppl. Figure S1), the disappearance of (001) reflection peak suggested the reduction of GO into graphene sheets. Additionally, in the diffraction spectrum of MnFe_2_O_4_/GAs, peaks corresponding to the diffraction planes viz., (220), (311), (400), (511), and (440) [JCPDS‐10‐0319]^23^ were also seen. Both MnFe_2_O_4_/GAs and MnFe_2_O_4_ displayed similar diffraction pattern. The diffraction peaks of the two samples can be traced to a face‐centered cubic crystal structure, indicating a phase pure synthesis of MnFe_2_O_4_.

The functional groups in GO, MnFe_2_O_4_, and MnFe_2_O_4_/GAs were analyzed by FT‐IR spectroscopy, as shown Figure 1b. The characteristic peaks of GO appeared at 1730 cm^−1^ (stretching vibration of C=O), 1622 cm^−1^ (skeletal stretching vibrations of C=C), and 1100 cm^−1^ (C−O stretching vibrations). On the other hand, for MnFe_2_O_4_/GAs, the characteristic peak of GO at 1730 cm^−1^ shifted to 1558 cm^−1^, implying that the MnFe_2_O_4_ particles were strongly adsorbed onto the GO surface by chemical reduction.^24^, ^25^, ^26^ From the FTIR spectrum of MnFe_2_O_4_/GAs, the vibration peaks of the most oxygen‐containing group disappeared, indicating that GO reduced during the hydrothermal and self‐assembly processes; this agrees with the XRD result.^27^

**Figure 2:**
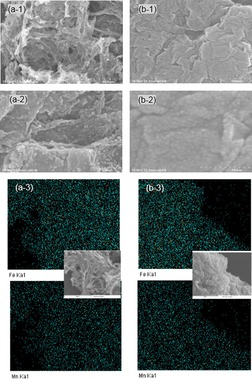
SEM images of MnFe_2_O_4_/GAs (a‐1, a‐2) and MnFe_2_O_4_/rGO (b‐1, b‐2), and EDX elemental maps corresponding to SEM images (a‐3, b‐3).

Figure 2 shows the SEM images and EDX elemental maps of MnFe_2_O_4_/GAs and MnFe_2_O_4_/rGO. For MnFe_2_O_4_/GAs, a 3D graphene aerogel framework with a continuous macroporous structure is clearly seen in Figure 2a. The graphene sheets were interconnected together forming a corrugated structures (Suppl. Figure S2). The MnFe_2_O_4_ nanoparticles were uniformly dispersed over the graphene aerogel matrix. On the other hand, the SEM images of MnFe_2_O_4_/rGO exhibit uniform‐sized MnFe_2_O_4_ nanoparticles formed on the graphene surface, as shown in Figure 2b. In addition, the elemental maps of both the catalysts revealed uniform distributions of Mn and Fe, and the elemental spectra showed that the Mn/Fe ratio was close to the theoretical loading ratio (Suppl. Figure S3).

The 3D‐structured MnFe_2_O_4_/GAs exhibited a high BET surface area of 169 m^2^ g^−1^ with an average pore size of 3.6 nm, whereas MnFe_2_O_4_/rGO had a BET surface area of 158 m^2^ g^−1^ with an average pore size of 5.03 nm, as measured by nitrogen adsorption (Suppl. Figure S4). Catalyst pore sizes of 2–10 nm range is known to be preferable for electrochemical applications because not only do these pores increase the active reaction sites, they also decrease mass‐transfer resistance.^28^ The nitrogen adsorption‐desorption isotherms MnFe_2_O_4_/GAs and MnFe_2_O_4_/rGO were observed to be type IV (according to IUPAC classification), indicating the presence of mesopores,^29^ which was consistent with pore size analysis. These isotherms showed H3 hysteresis loops, suggesting that slit shaped pores were formed by the aggregation of nonuniform sized and/or shaped graphene nanosheets.

### 2.2 Electrocatalytic Properties of MnFe_2_O_4_ NPs, MnFe_2_O_4_/rGO, and MnFe_2_O_4_/GAs

**Figure 3:**
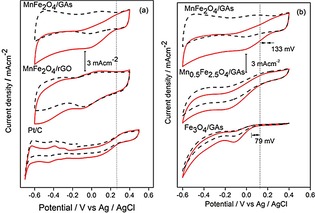
CV curves of MnFe_2_O_4_/GAs, MnFe_2_O_4_/rGO, and Pt/C (a) and Mn_x_Fe_3−x_O_4_/GAs (b) in O_2_ (solid) and N_2_ (dashed) saturated 0.1 M KOH electrolyte at a scan rate of 20 mVs^−1^.

The CV curves of MnFe_2_O_4_/rGO, MnFe_2_O_4_/GAs, and Pt/C obtained in O_2_‐ and N_2_‐saturated 0.1 M KOH aqueous solution are shown in Figure 3a. The reduction peak potentials of MnFe_2_O_4_/GAs and MnFe_2_O_4_/rGO appeared at −0.01 V and −0.1 V, respectively, indicating a considerable positive potential shift from MnFe_2_O_4_/rGO to MnFe_2_O_4_/GAs, and thus, a higher catalytic efficiency with a reduced overpotential of MnFe_2_O_4_/GAs for the ORR. In addition, ORR onset potentials of MnFe_2_O_4_/GAs and commercial Pt/C appeared at a similar position, suggesting that the catalytic activity of MnFe_2_O_4_/GAs is comparable with that of the Pt/C catalyst. In addition, the CV curves of Mn_x_Fe_3−x_O_4_/GAs with different *x* values are shown in Figure 3b. The results indicated that the presence of Mn oxide increased the ORR activity, mainly owing to the facilitation of the adsorption of oxygen by the corresponding redox of Mn oxide.^17^ Among the studied Fe/Mn ratios, the more positive ORR onset potential was observed for Fe/Mn=2/1.

**Figure 4:**
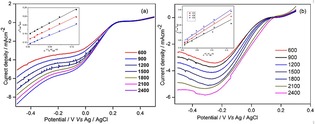
ORR polarization plots of MnFe_2_O_4_/GAs (a) and MnFe_2_O_4_/rGO (b) at different rpm in O_2_‐saturated 0.1 M KOH (insets: Koutecky‐Levich plots at different potentials) at a scan rate of 20 mV s^−1^.

The ORR kinetics of the MnFe_2_O_4_/GAs and MnFe_2_O_4_/rGO catalysts were examined by rotating disc electrode (RDE) measurements with different electrode rotation rates. The half‐wave potential of MnFe_2_O_4_/GAs at 900 rpm was 0.09 V, which was shifted positively as compared to that of MnFe_2_O_4_/rGO (−0.01 V), further indicating enhanced ORR activity of MnFe_2_O_4_/GAs probably due to its 3D structure. The insets in Figure 4a and 4b show the Koutecky‐Levich (K‐L) plots at different potentials. The linearity and parallel profiles revealed that the ORR on the catalyst surface of the electrode occurred by the first‐order kinetics and a similar number of electron transfer, respectively.^18^ From the slope of the K‐L plots, the number of electrons transferred on the catalysts was estimated to be ∼3.73 for MnFe_2_O_4_/rGO and ∼3.97 for MnFe_2_O_4_/GAs, suggesting a 4–e^−^ transfer process of ORR as similar to the 4–e^−^ process of ORR on Pt/C.^2^

**Figure 5:**
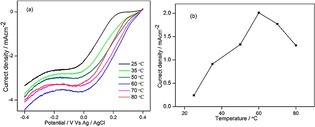
LSV of MnFe_2_O_4_/GAs in O_2_ saturated 0.1 M KOH at 900 rpm under different temperatures (a), and current density at 0.2 V vs. temperature (b).

Figure 5a presents the ORR polarization curves of the MnFe_2_O_4_/GAs catalyst at 900 rpm with different temperatures from 25 to 80 °C. The current density measured at 0.2 V increased with temperature up to 60 °C and then decreased, as shown in Figure 5b. According to Arrhenius equation, the ORR rate enhances with temperature. However, gaseous oxygen needs to be dissolved in an aqueous KOH solution before it is used in the ORR; therefore, a high temperature adversely affected the ORR rate because the solubility of oxygen in water decreased with temperature.

**Figure 6:**
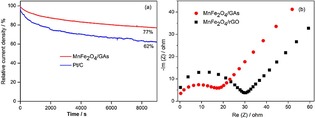
Chronoamperometric responses of MnFe_2_O_4_/GAs and Pt/C in O_2_‐saturated 0.1 M KOH at 0.1 V (a) and Nyquist plots of urea/O_2_ fuel cell with MnFe_2_O_4_/GAs and MnFe_2_O_4_/rGO cathode catalysts from 10 Hz to 5 MHz frequency.

The stabilities of MnFe_2_O_4_/GAs and Pt/C were studied by chronoamperometric measurements at a constant potential of −0.1 V, as shown Figure 6a. The MnFe_2_O_4_/GAs showed a slower current decay than the commercial Pt/C did. MnFe_2_O_4_/GAs retained 77 % of its initial current density after 150 min of continuous running. The deactivation of Pt/C in an alkaline solution is known to occur by the formation of Pt hydroxide on its surface.^30^

Electrochemical impedance spectroscopy measurement was carried out to further examine ORR on both MnFe_2_O_4_/GAs and MnFe_2_O_4_/rGO catalysts, as shown in Figure 6b. From the Nyquist plots, the charge transfer resistance was estimated from the diameter of the semicircle. The charge transfer resistance of MnFe_2_O_4_/rGO was 30.5 Ω cm^2^, and decreased to 17.5 Ω cm^2^ for MnFe_2_O_4_/GAs, further indicating that the MnFe_2_O_4_/GAs catalyst exhibited better charge‐transfer kinetics towards ORR.

### 2.3 Performances of uUea/O_2_ Fuel Cells with MnFe_2_O_4_/rGO and MnFe_2_O_4_/GAs

**Figure 7:**
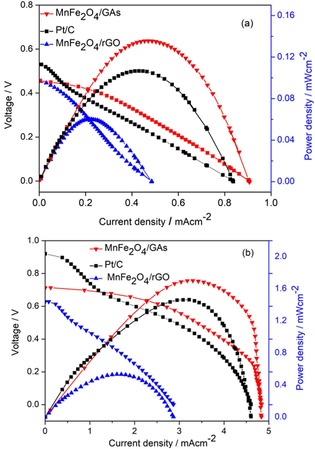
Performances of urea/O_2_ fuel cells with various cathode materials (MnFe_2_O_4_/rGO_,_ MnFe_2_O_4_/GAs, and Pt/C) in 0.33 M urea in 1.0 M KOH as an anolyte and humidified O_2_ as a catholyte at 25 °C (a) and 60 °C (b).

Urea/O_2_ fuel cells were fabricated using MnFe_2_O_4_/rGO, MnFe_2_O_4_/GAs, and Pt/C as cathode materials, separatively. The *I‐V* polarization and power density curves of these cells with 0.33 M urea in 1.0 M KOH feed as an anolyte and dissolved O_2_ bubbled as a catholyte at different temperatures are shown Figure 7. The best fuel cell performance was observed for the MnFe_2_O_4_/GAs cathode catalyst, mainly because of its mesoporous 3D network structure with a high BET surface area, as discussed earlier. MnFe_2_O_4_/GAs exhibited an OCV of 0.713 V and a maximum power density of 1.7 mw cm^−2^ at 60 °C, which was even higher than that of commercial Pt/C.

## 3 Conclusions

Manganese ferrite decorated on a graphene aerogel was synthesized and studied as a cathode catalyst for a urea/O_2_ fuel cell. GO was reduced, and MnFe_2_O_4_ nanoparticles were deposited on the highly porous 3D network‐structured MnFe_2_O_4_/GAs composite materials. The MnFe_2_O_4_/GAs catalysts exhibited a distinct electrocatalytic activity toward ORR, which was higher than that of MnFe_2_O_4_/rGO, with an enhanced stability, possibly because of its mesoporous 3D network structure with a high BET surface area. The MnFe_2_O_4_/GAs exhibited an OCV of 0.713 V and a maximum power density of 1.7 mw cm^−2^ at 60 °C, which was even higher than that of the commercial Pt/C. The results demonstrated that the 3D structured graphene aerogel‐supported MnFe_2_O_4_ can be a promising ORR catalyst.

## Experimental Section

### Synthesis of Graphene Oxide

Graphene oxide (GO) was synthesized from graphite flakes by a modified Hummers process.^31^ Briefly, 3.0 g of graphite flakes was added into 400 mL of concentrated H_2_SO_4_/H_3_PO_4_ (9 : 1). Then, 18 g of KMnO_4_ (18.0 g) was added and stirred for 12 h at 50 °C. After the reaction completed, it was cooled and poured into an ice water (400 mL) containing 6 mL of 30 % H_2_O_2_. The final product was centrifuged, washed to remove excess acid, and freeze‐dried at −60 °C for 72 h.

### Synthesis of Mn_x_Fe_3−x_O_4_‐Decorated Graphene Aerogel

Mn_x_Fe_3−x_O_4_ was impregnated into a graphene aerogel using metal salts and GO as precursors and hydrazine monohydrate as a reducing agent.^20^ First, 140 mg of GO was dispersed in 90 mL of deionized (DI) water by sonication for 30 min. To this suspension, stoichiometric amounts of Fe(Cl)_3_ ⋅ 6H_2_O and MnCl_2_ ⋅ 4H_2_O were added (Suppl. Table S1). The mixture was neutralized using 5 M NaOH, followed by the addition of 2 mL of hydrazine monohydrate as a reducing agent and stirred continuously for 30 min. It was then transferred to a 100‐mL autoclave reactor and kept at 180 °C under stationary condition for 12 h. The resulting black hydrogel was freeze‐dried to obtain graphene aerogel‐supported manganese ferrite oxides (Mn_x_Fe_3−x_O_4_/GAs).

MnFe_2_O_4_ nanoparticles (MnFe_2_O_4_ NPs) were also synthesized by reducing the precursor salts with hydrazine as described above. MnFe_2_O_4_ supported on reduced GO (MnFe_2_O_4_/rGO) was prepared by mixing MnFe_2_O_4_ NPs with GO suspention and reducing the mixture with hydrazine, as described elsewhere.^32^

### Preparation of Electrodes and Urea/O_2_ Fuel Cell Testing

The as‐prepared Mn_x_Fe_3−x_O_4_/GAs and MnFe_2_O_4_/rGO catalyst powders and the commercial Pt/C (20 %, E‐TEK) powder were dispersed in 5 % Nafion solution in isopropanol, respectively, and sonicated for 30 min. The resulting inks were coated on a glassy carbon electrode with a loading of 20 μg cm^−2^ and the electrochemical properties were measured. The catalyst ink was also coated on a 5.0‐cm^2^ carbon paper to prepare the cathode with a catalyst loading of 1 mg cm^−2^, and the anode was prepared using a commercial Ni/C (20 %, E‐Tek) with the same loading. An anion exchange membrane (AEM; Fumasep FAA‐3‐PK‐130, Germany) was used as a polymer electrolyte separating the anode and cathode compartments. Membrane electrode assemblies (MEAs) for fuel cell tests were fabricated from both the electrodes and AEM by hot‐pressing. A single‐cell bipolar plate was set using graphite with serpentine flow channels. A urea solution of 0.33 M in 1 M KOH was pumped into the anode side by a peristaltic pump at 2 mL min^−1^, and humidified O_2_ was supplied to the cathode.

### Analysis

The crystallographic phases and structures of the samples were examined using an X‐ray diffraction analyzer (XRD, Rigaku D/MAX‐2002, Japan) with Cu K_α_ radiation with a wavelength of 1.5406 Å by scanning the samples in the 2Θ range of 5° to 80° at a rate of 2° min^−1^. The morphology of the samples was studied by scanning electron microscopy (SEM, Hitachs‐4700, Japan). Functional groups in the powder were analyzed using a Fourier transform infrared (FTIR) spectrometer (Bruker, Saarbrucken, Germany). The Brunauer‐Emmett‐Telle (BET) surface area was measured from nitrogen adsorption and desorption isotherms, which were recorded at 77 K after degassing the analyte at 250 °C with a surface area analyzer (Micromeritics ASAP 2020, USA).

The ORR catalytic activities of the prepared samples were measured by cyclic voltammetry (CV), chronoamperometry (CA), and linear sweep voltammetry (LSV) by using a potentiostat (Biologic Sp‐240) and a rotating ring disc electrode apparatus (RRDE‐3A, ALS Company, Japan) with a three‐electrode configuration. A glassy carbon‐supported active material was used as the working electrode, Ag/AgCl filled with saturated KCl was used as the reference electrode, and Pt wire was used as the counter electrode. CV measurements were carried out with a supply of oxygen, while background current was collected by bubbling nitrogen. All current densities were normalized to the respective surface areas.

## Conflict of interest

The authors declare no conflict of interest.

## Supporting information

As a service to our authors and readers, this journal provides supporting information supplied by the authors. Such materials are peer reviewed and may be re‐organized for online delivery, but are not copy‐edited or typeset. Technical support issues arising from supporting information (other than missing files) should be addressed to the authors.

Supplementary
