# Supplementary material for: Synthesis and Evaluation of Graphene Aerogel‐Supported MnxFe3−xO4 for Oxygen Reduction in Urea/O2 Fuel Cells
Source: ChemistryOpen. 2019 May 14;8(5):615–20. doi: 10.1002/open.201900105 (PMC6515475; doi:10.1002/open.201900105)
Supplement: Supplementary file 1 — Supplementary [file OPEN-8-615-s001.pdf]

Open Access
ChemistryOPEN

Supporting Information

© Copyright Wiley-VCH Verlag GmbH & Co. KGaA, 69451 Weinheim, 2019

Synthesis and Evaluation of Graphene Aerogel-Supported $Mn_xFe_{3-x}O_4$ for Oxygen Reduction in Urea/O_2 Fuel Cells

Keyru Serbara Bejigo, Bang Ju Park, Ji Hyeon Kim, and Hyon Hee Yoon*©201x The Authors. Published by Wiley-VCH Verlag GmbH & Co. KGaA.
This is an open access article under the terms of the Creative Commons Attribution License, which permits use, distribution and reproduction in any medium, provided the original work is properly cited.

Table S1. Metal precursor's concentration during synthesis of $Mn_xFe_{3-x}O_4$ on graphene aerogel.

sample	$FeCl_3.6H_2O$ (M)	$MnCl_2.4H_2O$(M)	n (Fe^{3+}/Mn^{2+})
$MnFe_2O_4$/GAs	0.0267	0.013	2:1
$Mn_{0.5}Fe_{2.5}O_4$/GAs	0.033	0.006	5:1
Fe_3O_4/GAs	0.04	-	-

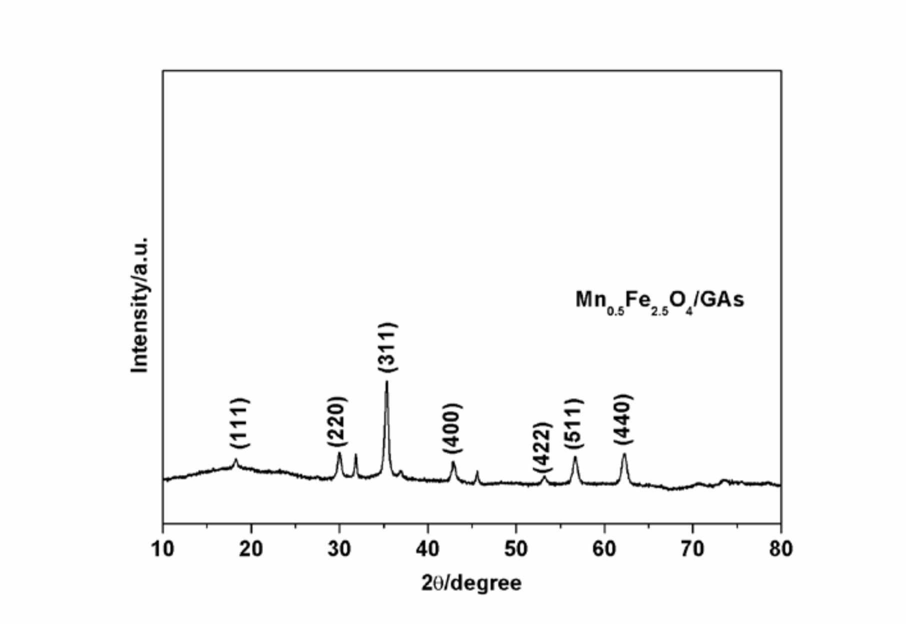

Fig. S1. XRD patterns (a) and FTIR spectra (b) of GO, $MnFe_2O_4$, and $MnFe_2O_4$/GAs.

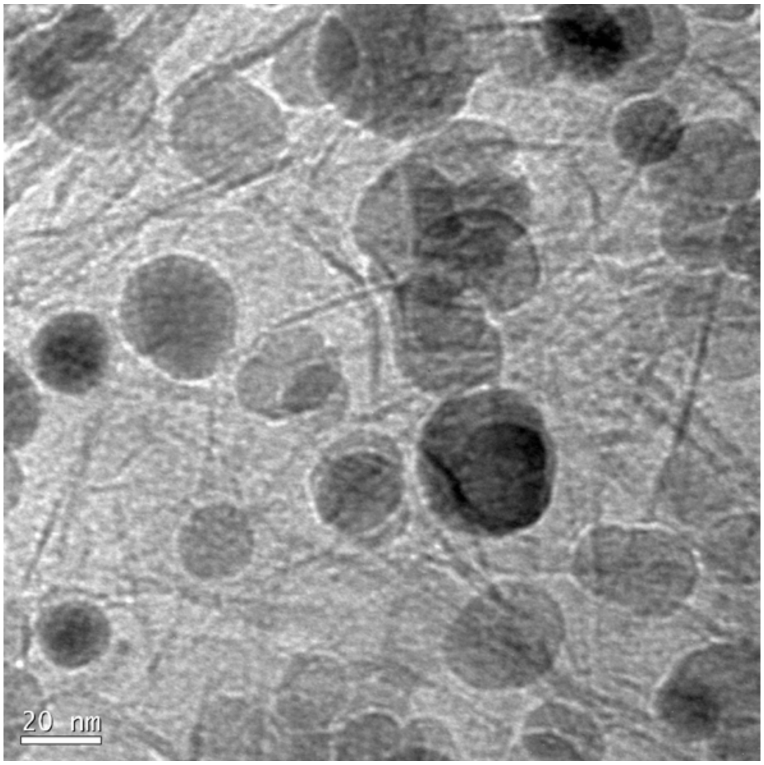

Fig. S2. TEM image of $MnFe_2O_4$/GAs.

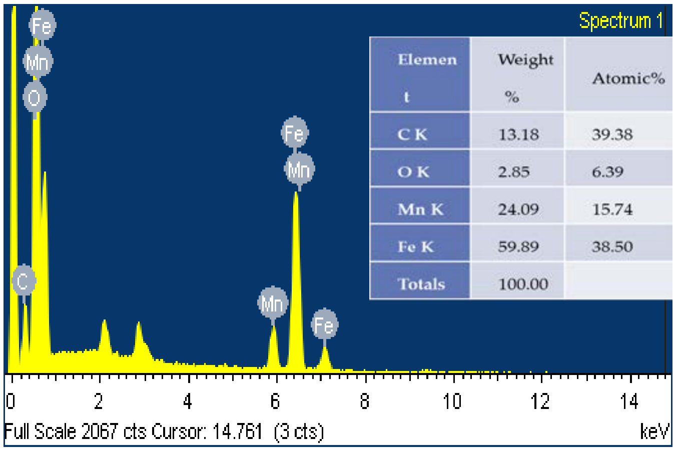

Fig. S3. EDX spectra of $MnFe_2O_4$/GAs (inserts: elemental compositions)

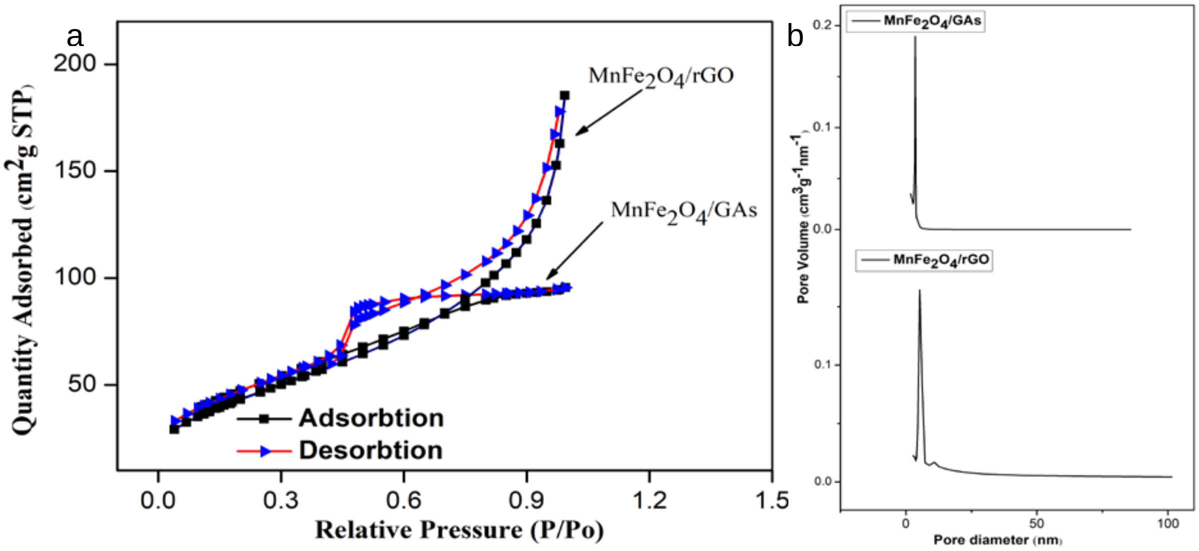

Fig. S4. Nitrogen adsorption and respective desorption isotherms (a) and pore size distribution (b) of $MnFe_2O_4$ /GAs and $MnFe_2O_4$ /rGO.

Table. S2. Performance comparison of urea fuel cells with different electrodes.

Anode	Cathode	Fuel and electrolyte Concentration	Max power density (mW/cm^2)	References
Ni/C	Pt/C	0.33 M urea in 1 M KOH @ 60 °C	1.4	[1]
Ni/C	MnO_2	1 M urea in 1 M KOH @50 °C	1.7	[2]
Ni/CNT	Pt/C	1 M urea in 3 M KOH	1.6	[3]
NiMn	Pt	0.5 M urea in 1 M KOH	3.6×10^{-3}	[4]
Ni/graphene	Pt/C	0.33 M urea in 1 M KOH	4.06×10^{-3}	[5]
Ni/C	$MnFe_2O_4$/ GAs	0.33 M urea in 1 M KOH @60 °C	1.7	This work

[1] W. Xu, H. Zhang, G. Li, Z. Wu, Sci. Rep. 4 (2014) 5863.

[2] R. Lan, S. Tao, J.T.S. Irvine, Energy Environ. Sci. 3 (2010) 438.

[3] H. Zhang, Y. Wang, Z. Wu, D.Y.C. Leung, J. Power Sources. 363 (2017) 61.

[4] N.A.M. Barakat, M. Alajami, Z.K. Ghouri, S. Al-meer, Nanomaterials, 8(2018) 338.

[5] A. Yousef, M.H. El-newehy, S.S. Al-deyab, N.A.M. Barakat, Arab. J. Chem. 10 (2017) 811.
